# High Acceptability of an Orally Dispersible Tablet Formulation by Children

**DOI:** 10.3390/children8030194

**Published:** 2021-03-05

**Authors:** Leonie Wagner-Hattler, Klara Kiene, Julia Bielicki, Marc Pfister, Maxim Puchkov, Jörg Huwyler

**Affiliations:** 1 Department of Pharmaceutical Sciences, Division of Pharmaceutical Technology, University of Basel, 4056 Basel, Switzerland; leonie.hattler@gmail.com (L.W.-H.); klarakiene@gmail.com (K.K.); maxim.puchkov@unibas.ch (M.P.); 2 Pediatric Pharmacology and Pharmacometrics, University Children’s Hospital (UKBB), University Hospital Basel, 4056 Basel, Switzerland; julia.bielicki@ukbb.ch (J.B.); Marc.Pfister@ukbb.ch (M.P.)

**Keywords:** oral drug administration, clinical trial, children, orally dispersible tablets, palatability

## Abstract

There is a high unmet medical need for child-appropriate oral dosage forms. The acceptability of a novel placebo orally dispersible tablet formulation (pODT) was therefore evaluated. Monolithic tablets contain an inorganic calcium carbonate/calcium phosphate carrier material as the main excipient. They were assessed in a cross-sectional acceptability study. The 40 child participants were between 2 to 5 years and 6 to 10 years old. One pODT with 5 mm diameter was administered to each participating child by placement on the tongue or into the buccal cavity. Parents were asked to complete a questionnaire together with the study personnel. The spontaneous reactions of the children were recorded. The ease of administration and children’s acceptance of the tablet was rated by research staff on a 4-point acceptability scale and by parents on a 5-point Likert scale. The older subjects answered how they had liked the pODT by pointing to the appropriate face of a Facial Hedonic Scale. pODT had very high acceptability as 93% of parents, and all questioned children reported the formulation to be acceptable or very acceptable. Staff reported administering pODT in these children without problems. None of the children showed distress on receipt of pODT. We conclude that the proposed child-friendly dosage form provides a convenient option for oral drug administration and is expected to enhance drug-adherence in pediatric patients.

## 1. Introduction

Oral drug administration is the primary choice in the community-based management of any childhood disease requiring systemic treatment [1]. However, despite offering advantages for stability and handling, very few solid oral dosage formulations designed for young children are available. Most of the drugs on the market are authorized only for adult use [2]. The latter is a known problem since a medication’s taste and the ability of children to swallow their medicine may significantly influence the selection of a drug and the prescribing practice [3]. Thus, medication palatability is essential for patient acceptance, therapeutic compliance, and successful therapy outcome [4,5]. In an attempt to adjust doses, pediatricians need to prepare and administer unlicensed formulations by manipulating adult dosage forms (“off-label use”), which includes splitting or crushing tablets, opening capsules, dispersing tablets or capsules in liquids, and taking proportions of them to adjust doses, cutting suppositories, or applying injectable solutions by other routes. However, such manipulations inevitably influence the safety and eventually bioavailability and pharmacokinetics of a medicinal product [6]. Manufacturers are therefore requested to provide information on the consequences of such manipulations, perform pediatric investigations with the products intended for pediatric use, and develop child-appropriate dosage forms and medicines [6]. However, formulating medication for children is challenging since pediatric formulations have to allow accurate dosing and patient acceptance [7]. Different age classes and interregional differences make it necessary to produce different types of galenical formulations in several drug concentrations, flavors, and colors [7,8]. Therefore, far, there is not much known about the acceptability of different dosage forms, the number of dose units, administration volumes, dosage form size, taste, and acceptability and safety of excipients concerning age and development status of the child [6].

Based on our previous research, orally dispersible tablets are very promising drug formulations [9]. The palatability of an oral dispersible tablet formulation based on Functionalized Calcium Carbonate (FCC) was already assessed in adults, showing good acceptability, pleasant taste due to the addition of aroma and sweeteners, and no bad sensations during administration [10]. It has been previously reported that the excipient FCC is tasteless. Given these encouraging results, the question arises whether the palatability in children is as positive as in adults since it is not possible to transfer the findings from adults directly to children due to different taste and mouthfeel sensations [6,11]. Therefore, far, there has not been much systematic methodological research on how to evaluate the taste and mouthfeel of age-appropriate formulations in pediatric patient populations [12].

As such, the present study’s primary goal was to evaluate the acceptability of a pODT formulation in terms of palatability, based on insoluble carrier material, for administration to preschool and school-age children. The secondary objective was to apply a comprehensive assessment tool collecting parental, child, and staff acceptability feedback.

## 2. Materials and Methods

### 2.1. Placebo Orally Dispersible Tablets (pODT)

pODTs [13] with a diameter of 5 mm, containing FCC (OmyaPharm 500 OG, LOT OG/0001/53J; Omya, Oftringen, Switzerland), croscarmellose sodium (Ac-Di-Sol^®^ SD 711NF; FMC International, Cork, Ireland), food-grade orange aroma (Givaudan, Vernier, Switzerland), citric acid (citric acid monohydrate; Carl Roth, Karlsruhe, Germany), sodium hydrogen carbonate (Hänseler, Herisau, Switzerland), and a cyclamate/saccharine mix (Sodium Saccharin/Sodium Cyclamate; Sanaro, Vouvry, Switzerland), were prepared by University of Basel, Division of Pharmaceutical Technology [10]. FCC is tasteless. Aroma and sweeteners were not added to mask the taste but to enhance palatability. In brief, granules for tablet manufacturing were produced by roller compaction (i.e., dry granulation) and subsequent blending. The composition of pODTs is provided as Supplementary Materials and is available from authors on request.

The tablets were compacted with Styl’One Classic compaction simulator (Medel’Pharm, Lyon, France). A Euro B 5 mm flat punch tooling was used. The compressive force was set to 5 kN. Tooling lubrication with magnesium stearate (Hänseler) was carried out manually for each tablet before tablet compaction. The prepared tablets were certified as fit for human consumption by an appropriately qualified person. The relevant documentation and the certificates of manufacture and analysis are provided by the authors on request or downloaded from http://pharma-te.ch/odt_children/ (last accessed on 4 March 2021).

### 2.2. Setting and Participants

This single-center cross-sectional observational study included 40 children attending the University Children’s Hospital Basel (UKBB) outpatient surgical and fracture clinic. The children were between 2 years and ten years old, as this is the target group that we believe is most likely to benefit from a pODT formulation being available as an alternative to oral suspensions. The younger group (20 children) with age range between 2 and 5 years, the older volunteers (20 children) were 6 to 10 years old. Female and male volunteers were eligible for enrolment if they were 2 to 10 years of age, indicating their verbal assent to take part and for whom the parents provided their written informed consent. Volunteers were excluded for any of the following reasons: wearing fixed dental braces; suffering from injuries or inflammations affecting the oral cavity or throat; suffering from dysphagia, olfactory impairment, kidney impairment, or hypercalcemia; known allergy against medications; ongoing antibiotic treatment at the time of the study; known moderate to severe developmental delay as reported by the parents; participation in any other study within the 30 days preceding and during the study; if parents were unlikely to reliably complete the structured questionnaire or understand informed consent due to significant language barriers.

### 2.3. Study Procedures

An Institutional Ethics Committee approved the study protocol and consent forms on 21 August 2017. The study was registered at ClinicalTrials.gov (registration number 2017-01367) and the SNCTP (Swiss National Clinical Trials Portal registration number NCT03581799). The start of the study was 29 January 2018. The study was completed as planned and without adverse events or changes to the initial protocol on 12 March 2018.

Each study consisted of a ~30-min visit without follow-up visits. The participants were screened on days of routine appointments in the fracture and surgical outpatient clinic of the UKBB and approached during waiting times. If a child was identified to meet the inclusion criteria, designated and trained research staff obtained informed consent from the child’s legal guardian and verbal agreement.

### 2.4. Intervention and Assessment

**Figure 1:**
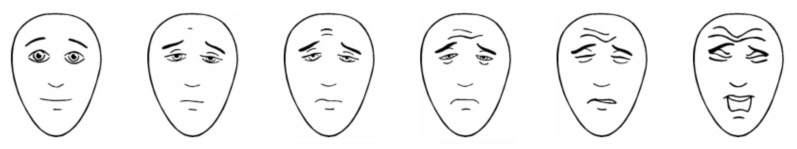
Facial hedonic scale (FHS) was used to evaluate the taste sensation of children between 6 to 10 years. The children reported the face that described their experience with the pODT best. The pointed-out face was then assigned a number by the staff ranging from 0 (“very good”) to 10 (“very bad”).

One pODT was placed onto the tongue or into the buccal cavity of a participating child. Parents were subsequently asked to complete a questionnaire together with the study personnel. A questionnaire developed for adult participants [10] and adapted to suit parental reporting of acceptability and palatability was used (Table 1). The questionnaire is available from authors on request. The children’s spontaneous reactions (e.g., positive comments, spitting out of pODT, crying) were observed and recorded by the staff to identify low acceptability. In addition, administration and children’s acceptance of the tablet were rated by research staff on a 4-point acceptability scale (from “child wants to take the pODT” to “refusal”) and parents on a 5-point Likert scale (from “very acceptable” to “completely unacceptable”) *(*Table 1). The older subjects (age 6 to 10 years) were questioned on how they had liked the pODT by pointing to the appropriate face of a Facial Hedonic Scale (FHS) (Figure 1) [14,15]. They were asked whether they would like to have a second tablet on another occasion, how fast the pODT disintegrated, and where residues were felt within the mouth. The latter answers were collected by the study personnel based on visual inspection. After finishing the assessment, children and parents were free to leave. Participants were allowed access to drinks during or following administration.

### 2.5. Statistical Analysis, Sample Size, and Demographic Characteristics

The objective of the statistical analysis was to assess palatability, which was the primary study variable. The secondary objective was time to disintegration in the oral cavity. Subjects were included in the palatability analyses if they satisfied the entry criteria and finished the evaluation. The required sample size to detect reported palatability of 80% of the pODTs (alpha 0.05, power 0.8) assuming a 50% palatability rate under the null hypothesis (i.e., indifference) is 20 patients per group based on the parentally reported outcome of acceptability (5 points Likert scale). In total, 40 children (2–5 years of age, *n* = 20; 6–10 years of age, *n* = 20) were included in the study. The age distribution of participating children was uniform, with a median age of 71.5 months (IQR 45.5–98.5, minimum 24 months, maximum 125 months). The median age of parents was 37.5 years (IQR 34–43, minimum 27, maximum 56). As expected, the median age of parents in the younger age group was lower (35 years, IQR 33–40.5) than in the older age group (40 years, IQR 34.5–44). The primary language of 63% of the parents was German or Swiss German, the locally spoken languages.

Each participant received one orange flavored, FCC-based pODT, which was placed onto the tongue or into the buccal cavity. Depending on the age group, palatability was assessed by parents and staff only or by additional child-appropriate questions. The questionnaire is available from authors on request.

### 2.6. Data Handling

All relevant non-questionnaire data were collected on project-specific case report forms (paper format) by trained study personnel. They served as the source documents. All paper-based study information (questionnaires and CRFs) was entered into a secure password-protected REDCap^TM^ database system (Vanderbilt University, Nashville, TN, USA) hosted at UKBB.

## 3. Results

Seventy-eight children were included in the present study. Thirty-eight children had to be excluded for several reasons: they did not appear at the hospital, or enough children in their age group were already included (17/78, 22%); their parents had not enough time to participate (3/78, 4%); their parents refused to participate (8/78, 10%); children did not give verbal assent (1/78, 1%); no reliable communication between staff and parents was possible due to language barriers (4/78, 5%); children met medical exclusion criteria (5/78, 6%).

The orally dispersible placebo carrier tablet had very high acceptability. The staff reported administering the pODT without any problems for almost all children (39/40, 98%). The remaining child was from the younger age group and spit out the tablet (see below). In 37/40 (93%) observed encounters, staff did not note any signs that pODT was not acceptable. In the remaining 3/40 (7%) younger children, staff pointed out some reluctance of taking the pODT (in 2 cases) or reported spitting out of the tablet (1 younger child). Most parents said the formulation to be acceptable or very acceptable (37/40, 93%). The only three exceptions were children from the younger age group where parents had a “neutral” impression. Parental acceptability was strongly related to parent gender, with mothers more likely to indicate that the pODT was very acceptable (25/29, 86%; fathers 6/11, 55%; *p* = 0.011). Parents whose children had previously received oral medications, 13/35 (37%), reported difficulties in administering conventional marketed child-appropriate formulations. The pODT was reported to be easier to administer by 27/35 (77%) of parents. However, since doses, size, and contained drugs of marketed drug formulations are unknown, no preference claims can be made. None of the children showed distress on receipt of the tablet. Using the FHS (Figure 1), all children of the older subpopulation (20/20, 100%) rated the palatability of the pODT between 0 (17 children) and 2 (3 children). Moreover, 80% (16/20) reported that they would agree to take a second pODT on another occasion.

All children in the older age group were explicitly questioned about the performance of the pODT, in that they were asked to indicate complete disintegration of the tablet and answer questions about any residual material. Besides, some of the younger children spontaneously reported that the pODT had disintegrated. The median disintegration time in the older age group was 16s (IQR 12–28, min 6, max 75). In this group, 13/20 (65%) indicated some residual material after the disintegration of the pODT. In total, 12/20 children in the younger age group spontaneously indicated that the tablet had disintegrated (min 3 s, max 20 s), with some of these also stating or demonstrating some residual material, mostly on the tongue. pODT residues were distributed mainly on the tongue and within the cheek cavity, but neither were they detected in the corners of any child’s mouth, nor did any child try to get rid of pODT residues e.g., by spitting them out or communicating an urge to drink.

## 4. Discussion

When planning a clinical trial with children, it is necessary to adapt the methods to the child’s developmental stage and focus on practical and ethical considerations and limitations [16]. According to the EMA, palatability studies for children should be short, entertaining, easily understandable, and with as few variables as possible. The younger the children, the more critical it is to respect these principles. Moreover, it has to be kept in mind that younger children have difficulties communicating their feelings and preferences. Generally, the EMA considers children older than four years as capable to participate in palatability trials [6]. In agreement with these principles, participants were screened during waiting times on days of routine appointments. For participating children and parents, the palatability study did provide some distraction and entertainment. The duration of the intervention was 5 to 10 min, which was short enough to maintain the attention and focus of both younger and older children.

The children were divided into two subgroups (ages 2 to 5 and 6 to 10 years). One parent accompanied each child. It was already recommended to assess taste acceptance in children younger than five years by using the child’s spontaneous verbal judgments. Moreover, it was suggested to involve parents by asking about any discomfort or other observations concerning the acceptance of the study medication by their child, which would ensure a reliable outcome of a palatability study with young children [17,18]. Consequently, acceptance was judged based on the combined feedback from children, parents, and staff. The spontaneous verbal and non-verbal reactions of all children were noted by staff and parents. The combined assessment of parents and staff was helpful because there were some instances where parents indicated the administration was very acceptable, but in fact, signs of poor acceptability were observed by staff. Keeping in mind that there are difficulties for younger children to communicate their preferences between several formulations, the focus was on acceptance of the pODT’s palatability [17]. Since there is limited consensus on the age at which children can reliably communicate subjective impressions or use hedonic facial scales [19], only children older than six years were considered to express acceptability using an FHS as well as verbal judgment [20,21].

The used formulations are considered to be safe in their application. None of the children described an unpleasant mouthfeel. No adverse events were reported. There were no observations of coughing or gagging, and all pODTs disintegrated rapidly. The mean disintegration time measured in children between 6 and 10 years was comparable to that of adults (26 s vs. 22 s [10]). This shows that the formulation of such pODT is well suited for children even if it is assumed that the mouth and tongue movement during administration greatly influences the pODT’s disintegration time.

Concerning the limitations of the study, it should be mentioned that the placebo tablets are not a representative medicine as they do not contain a bitter agent. Furthermore, the size and shape of a tablet are factors, which were not evaluated in the present study. Indeed, the size and number of administered tables can create a significant challenge. The study was carried out in the presence of parents. Therefore, a potential bias introduced by the parents’ encouraging or hesitant non-verbal responses cannot be excluded.

## 5. Conclusions

The evaluated placebo orally dispersible tablet based on insoluble carrier material was highly acceptable for administration to pre-schoolers and school-age children. It remains to be elucidated if multiple orally dispersible placebo tablets could be used as an alternative to multi-particulate granules or pellets. Future studies will focus on preparing palatable inert carrier ODTs for drugs that are currently available only as bad-tasting liquid formulations or are unavailable as child-friendly formulations. These include frequently used drugs, such as antibiotics and steroids. Greater use of this or similar child-appropriate solid oral formulations could improve access to high-quality medicines, enhance drug-adherence, and as such benefit millions of pediatric patients worldwide.

## Figures and Tables

**Table 1:** Study design and assessment. The term ‘Acceptable’ is defined by the first two points on the Likert scales. Age group “younger”: 2–5 years of age, *n* = 20. Age group “older”: 6–10 years of age, *n* = 20.

Age Group	Assessment by	Question	Options
Younger and older	Staff	Spontaneous reaction Acceptance (4-point acceptability scale)	Positive or negative comments Crying Spitting out of pODT A child wants to take pODT Administration of pODT without problems Persuasive efforts necessary to administer pODT Refusal of pODT
Younger and older	Parent	Acceptance (5-point Likert scale) Comparison to known medication	Very acceptable Acceptable Neutral Unacceptable Completely unacceptable Easier administration Comparable ease of administration More difficult administration
Older	Child	Liking (Facial Hedonic Scale, FHS) The second pODT wanted Disintegration of pODT	Five faces representing a scale of 0 (very good) to 10 (very bad) Yes/No Time in seconds Residues of pODT in the mouth

## Supplementary Materials

Supplementary information is available from authors on request or can be downloaded from http://pharma-te.ch/odt_children/ (accessed on 4 March 2021). This includes tablet formulation composition, certificate of manufacture and analysis, and a CONSORT Check List and Flow Diagram.

## Trial Registration

ClinicalTrials.gov identifier: NCT03581799.

## Registration References

https://clinicaltrials.gov/show/NCT03581799, https://www.kofam.ch/en/snctp-portal/search/81410/study/42927 (last accessed 4 March 2021).
